# An update on advanced therapies for Parkinson's disease: From gene therapy to neuromodulation

**DOI:** 10.3389/fsurg.2022.863921

**Published:** 2022-09-23

**Authors:** Stephanie N. Serva, Jacob Bernstein, John A. Thompson, Drew S. Kern, Steven G. Ojemann

**Affiliations:** ^1^ School of Medicine, University of Colorado Anschutz Medical Campus, Aurora, CO, United States; ^2^ Department of Neurosurgery, University of Colorado Anschutz Medical Campus, Aurora, CO, United States; ^3^ Department of Neurology, University of Colorado Anschutz Medical Campus, Aurora, CO, United States

**Keywords:** movement disorders, Parkinson's disease, deep brain stimulation, gene therapy, advanced therapies, focused ultrasound

## Abstract

Advanced Parkinson's disease (PD) is characterized by increasingly debilitating impaired movements that include motor fluctuations and dyskinesias. At this stage of the disease, pharmacological management can result in unsatisfactory clinical benefits and increase the occurrence of adverse effects, leading to the consideration of advanced therapies. The scope of this review is to provide an overview of currently available therapies for advanced PD, specifically levodopa–carbidopa intestinal gel, continuous subcutaneous apomorphine infusion, radiofrequency ablation, stereotactic radiosurgery, MRI-guided focused ultrasound, and deep brain stimulation. Therapies in clinical trials are also discussed, including novel formulations of subcutaneous carbidopa/levodopa, gene-implantation therapies, and cell-based therapies. This review focuses on the clinical outcomes and adverse effects of the various therapies and also considers patient-specific characteristics that may influence treatment choice. This review can equip providers with updated information on advanced therapies in PD to better counsel patients on the available options.

## Introduction

Parkinson's disease (PD) is a progressive degenerative disorder of the nervous system, characterized by motor and nonmotor symptoms (1). Early in the disease course, medical management with oral medications, including dopaminergic and nondopaminergic options, is the first-line treatment. As the disease progresses, management with oral medications alone becomes increasingly more challenging and may be limited by medication-induced side effects. Advanced PD is characterized by increasingly debilitating impaired movements that include motor fluctuations and dyskinesias. At this stage of the disease, pharmacological management can be associated with unsatisfactory clinical benefits and/or produce adverse effects. Consequently, therapies including device-aided pharmacotherapies, ablative or lesioning procedures, and deep brain stimulation (DBS) are often considered. Device-aided pharmacotherapies improve symptoms through continuous dopaminergic medication delivery, while ablative procedures and DBS provide targeted ablation/stimulation of the motor circuit. This review provides an overview of currently available therapies for advanced PD that are FDA-approved, specifically levodopa–carbidopa intestinal gel (LCIG), radiofrequency (RF) ablation, stereotactic radiosurgery (SRS), MRI-guided focused ultrasound (MRgFUS), and DBS. Therapies in clinical trials or that are not FDA-approved will also be discussed including continuous subcutaneous apomorphine infusion (CSAI), novel subcutaneous carbidopa/levodopa formulations, gene-implantation therapies, and cell-based therapies. The landscape of treatment options for advanced PD is rapidly evolving, and head-to-head studies comparing the efficacy and safety of advanced therapies are lacking. In the following paragraphs, the clinical outcomes and adverse effects of the various advanced therapies will be discussed. Patient-specific characteristics can also impact treatment choice, and these factors will also be considered. This review can equip providers with updated information on advanced therapies to better counsel patients on the available treatment options.

## Infusion therapies

Infusion therapies for PD are applied on the rationale that continuous increased availability of striatal dopamine (DA) can significantly reduce motor fluctuations and the variability in the treatment response (2). Numerous clinical studies demonstrate that continuous dopaminergic stimulation downregulates motor fluctuation and dyskinesia compared to intermittent dopaminergic stimulation. LCIG and CSAI are the only available infusion therapies on the market at this time (3). CSAI is currently available in Europe and is under review for use in the United States. The safety and efficacy of continuous subcutaneous levodopa are also being explored (4).

### Levodopa–carbidopa intestinal gel

LCIG infusion, also known as carbidopa/levodopa enteral suspension and by the trademark names of Duopa™ and Duodopa™, is a suspension gel containing carbidopa–levodopa in a standard 1:4 ratio that aims to deliver the drug directly to the proximal small intestine (5). The drug is delivered *via* a portable pump that is connected to a percutaneous endoscopic transgastric jejunostomy (PEG-J) (6). Pharmacokinetic studies have demonstrated that LCIG results in lower variability and fluctuations in levodopa plasma concentrations compared to oral levodopa (7). Several studies have evaluated the safety and efficacy of LCIG and are described below.

#### Clinical outcomes of LCIG

In the first double-blind, placebo-controlled trial of LCIG, Olanow et al. assessed the efficacy and safety of LCIG (8). Over 12 weeks, the off time was reduced by 1.91 h and the on time without troublesome dyskinesia (TSD) was increased by 1.86 h from a baseline of 6.3 and 8.7 h, respectively. In a multicenter, prospective, open-label study by Buongiorno et al., 72 patients with PD were treated with LCIG and followed for a mean observation time of 22 months. There was a significant reduction of 3.8 h from a baseline of 6.8 h in the mean off time per day (9). In an international, prospective, open-label LCIG study, PD patients received LCIG monotherapy and experienced a 4.4 h reduction in off time from a baseline of 6.8 h, a 65.6% improvement. On time without TSD was significantly increased by 4.8 h from a baseline of 7.67 h, a 62.9% improvement (10). Patients who completed this study were eligible for enrollment in a phase III multinational study. A total of 262 patients contributed to the safety dataset, and 86 patients were included in the efficacy subset. The mean total exposure to LCIG was 4.1 years. Patients in the efficacy cohort experienced a significant decrease of nearly 4 h in off time per day from prior to initial LCIG infusion to the study endpoint. Mean daily on-time hours without TSD were increased by approximately 4 h. Unified Parkinson's Disease Rating Scale (UPDRS) II and III scores were significantly improved by 3.03 and 4.77 points, respectively, from prior to initial LCIG infusion to study baseline (11). The GLORIA registry evaluated the 24-month safety and efficacy of LCIG treatment, and 258 patients enrolled by 75 movement disorder centers were used for this registry. At the last visit, treated patients showed a 3.9 h reduction in off time from a baseline of 6.0 and 1.1 h reduction in on time with dyskinesia from a baseline of 4.3 h (12). The GREENFIELD observational study assessed the effect of LCIG therapy on motor and nonmotor symptoms. There was an improvement in mean UPDRS-IV Item 39 (daily off time) of 55% and 50% at second and third follow-up visits, respectively (13).

#### Safety and adverse events of LCIG

Despite being an efficacious alternative to oral therapies, the high rate of adverse events (AEs) associated with LCIG must be considered. In total, 55%–95% of patients experience AEs, the majority being mild to moderate (8, 10–13). The most common AEs were device- or procedure-related, including procedural pain, abdominal pain, and postoperative wound infection (14). Serious AEs (SAEs) range from 27% to 53%, and the most common SAEs include peritonitis, pneumonia, and abdominal pain (10–15). The annual rates of percutaneous endoscopic gastrostomy and jejunal tube replacements are around 16% and 30%, respectively, as reported by long-term studies (11).

#### Patient considerations for LCIG

LCIG is a suitable choice for patients with levodopa-responsive PD with motor fluctuations that are not adequately controlled with oral therapy. There are no age limitations associated with LCIG therapy, although this treatment is cautioned in patients with severe cognitive decline and/or severe dopaminergic psychosis. Nevertheless, this therapy has been successfully applied to some patients with dementia who are unable to receive other advanced therapies (16). Because the initiation of LCIG therapy requires a PEG-J implantation procedure, the presence, or history of, gastrectomy/previous gastroenteroanastomosis must be carefully assessed. If the patient is unable to manage the pump adequately, LCIG also requires the availability of a responsible caregiver who can be taught to operate the pump (17).

### Continuous subcutaneous apomorphine infusion

Apomorphine is a dopamine agonist with a mixed affinity for D1 and D2 receptors and an affinity for serotonergic and alpha-adrenergic receptors (2). While the motor efficacy of apomorphine is similar to that of levodopa, apomorphine cannot be administered orally due to its low oral bioavailability (18, 19). Subcutaneous infusions have a similar pharmacokinetic profile to the intravenous route. Compared with intermittent subcutaneous injections, CSAI has a longer apparent plasma half-life and simulates the physiological stimulation of striatal neurons (20–22).

#### Clinical outcomes of CSAI

In the OPTIPUMP cohort study, the efficacy and safety of CSAI were assessed. The total UPDRS score showed significant improvement in all patients at 6 months. UPDRS-III decreased in the on-medication state by 16.3% (23). In the first prospective, randomized, placebo-controlled trial to investigate the efficacy and safety of apomorphine subcutaneous infusion in PD patients, a daily off time reduction of 1.89 h from a baseline of 6.69 h was seen in treated patients, while there was an increase of 1.97 h in on time without TSD from a baseline of 8.52 h (24). In a retrospective study, 230 patients were treated with CSAI over 10 years. In this cohort, the daily off hours were reduced from 5.4 to 1.2 h (25).

#### Safety of CSAI

Almost all patients on CSAI experienced infusion site reactions, with subcutaneous nodules being the most common form. The majority of reactions are considered mild to moderate, although there have been reports of severe cases of necrosis and abscess formation (2, 26). Emesis and hypotension can also occur, although these side effects are more common with intermittent injection than CSAI (27, 28). Rare cases of hemolytic anemia have also been reported, the mechanism of which remains unclear (29). Additionally, CSAI may fail to resolve impulse control disorders (ICDs) for PD patients with existing ICDs, or potentially induce new ICDs (30).

#### Patient considerations for CSAI

Patient suitability for CSAI is similar to that of LCIG in that it is an appropriate option for patients with levodopa-responsive PD with motor fluctuations not adequately controlled with oral therapy. CSAI is less invasive than LCIG in that initiation of treatment does not require a surgical implantation procedure. CSAI also requires the availability of a caregiver to aid with the infusion pump should the patient be unable to manage it on their own adequately. Given the potential risk of worsening neuropsychiatric symptoms, specifically ICDs, patients with these pre-existing conditions should be carefully monitored when placed on CSAI (30).

### Subcutaneous carbidopa/levodopa

The efficacy and safety of novel formulations of subcutaneous carbidopa/levodopa are also explored. ND01612 (NeuroDerm) is a novel formulation of levodopa/carbidopa that is administered subcutaneously *via* a pump. In a 28-day open-label study, 33 PD patients were randomized to treatment with either a 24-h infusion or a 14-h waking day infusion. The overall off time was reduced by 2 h from a baseline 5.3 h, with a larger reduction in the 24-h group (2.8 compared to 1.3). Eight patients in the 24-h group experienced complete resolution of the off time. The on time without TSD was increased by 3.3 h from a baseline of 8.9 h, and the on time with TSD was reduced by 1.2 h overall from 1.9 h at baseline. The 24-h infusion group experienced a −19.1 mean change in the UPDRS-III score from baseline. The most common AEs were infusion site reactions (4).

Foslevodopa/foscarbidopa (ABBV-951) is a subcutaneous levodopa/carbidopa prodrug formulation that is in development for treating the motor complications of advanced PD. A phase 1, single-blind study demonstrated that foslevodopa/foscarbidopa infusion provides stable levodopa and carbidopa exposures compared to oral dosing. The safety and tolerability of the infusion were also assessed, and the safety profile was determined to be favorable. Five of the 28 subjects had treatment-related AEs, including infusion site pain, dizziness, dyskinesia, paresthesia, and euphoric mood. Only one adverse event of anxiety was considered severe, while all other AEs were mild or moderate in severity (31).

## Ablative therapies

Ablative therapies for neurological disorders aim to destroy a targeted volume of brain tissue (32). Here, we discuss three ablative therapies used in the treatment of PD: RF ablation, SRS, and MRgFUS. Radiofrequency ablation has been largely supplanted by DBS and MRgFUS but is still performed in resource-limited areas, as the latter-mentioned therapies are technologically demanding and expensive.

### Radiofrequency ablation

Interstitial RF consists of creating a lesion through frictional heating of ionic oscillations of an intracranially placed electrode coupled to an RF generator. During the procedure, patients are awake, and the target is confirmed with a test stimulation. A lesion is created near the tip of the active electrode, which is where the greatest heating occurs. RF lesioning allows for the creation of distinct lesion borders with immediate results and intraoperative confirmation of symptom improvement (33, 34).

### Clinical outcomes and adverse events of RF

#### Ventral intermediate

Ablation of the ventral intermediate (VIM) nucleus of the thalamus has been shown to significantly improve tremors in patients with tremor-dominant Parkinson's disease (TDPD). A retrospective study by Jankovic et al. demonstrated that 86% of patients with PD experienced moderate to complete tremor improvement at a mean follow-up of 53 months (35). Most AEs with RF thalamotomy are transient and due to the perilesional edema that resolves with time. However, persistent adverse effects may occur, including ataxia, gait disturbances, dysarthria, and motor/sensory deficits (36). While RF thalamotomy can be performed bilaterally, the risk of dysarthria is significantly higher with bilateral lesions, so the procedure is generally only performed unilaterally (33).

#### Globus pallidus internus

Globus pallidus internus (GPi) ablation has also been shown to improve motor symptoms in PD patients (37). A randomized controlled trial demonstrated that patients receiving unilateral pallidotomy had a 32% improvement in the UPDRS-III score at 6-month follow-up compared to those randomized to medical therapy. Patients’ experienced improvements in tremor, rigidity, bradykinesia, gait, and balance after the procedure (38). Pallidotomy and thalamotomy carry similar cognitive and motor risks, although pallidotomy also carries the risk of visual disturbance (33). Although bilateral pallidotomy can be effective, the increased incidence of adverse effects must be considered against the marginal gain of performing the second side. A bilateral procedure is generally not recommended, especially considering the availability of other techniques/procedures (39).

#### Subthalamic nucleus

Ablation of the subthalamic nucleus (STN) has been explored for treating the motor symptoms of PD. A 43%–52% improvement in off-medication UPDRS-III scores has been reported in more recent trials of RF subthalamotomy. Adverse effects include contralateral dyskinesias and transient hemiballism (33).

### Patient considerations for RF

RF ablation allows for the creation of distinct lesion borders and intraoperative physiological confirmation of lesion creation. RF ablation provides immediate treatment effects, but the effects are not adjustable or reversible. Drawbacks include less predictability in the size and shape of the lesion. While adverse effects are generally mild and transient, the surgical risk of intracerebral hemorrhage must be considered (33). While used less commonly in high-income countries, ablative therapies requiring fewer resources such as RF remain a reasonable treatment strategy in emerging countries with limited resources (40).

### Stereotactic radiosurgery

SRS lesioning is a noninvasive procedure that uses computerized dosimetry planning and image-guided stereotaxy to deliver a single dose of ionizing radiation to an intracranial target volume. Different devices are used to deliver radiation, including GammaKnife® and linear accelerators (34).

### Clinical outcomes and adverse events

Gamma knife (GK) thalamotomy has been shown to improve motor function in patients with PD. The radiation dose used in GK thalamotomy ranges from 120 to 180 Gy, and it appears that doses less than 120 Gy do not provide clinical benefits (41). In a prospective study of unilateral gamma knife thalamotomy for patients with PD and essential tremor (ET), Ohye et al. reported an improvement in tremor scores (UPDRS-II and III), with 81.1% of patients evaluated as having good or excellent results at 24-month follow-up (42). A more recent prospective, single-blind study of unilateral GK thalamotomy for patients PD and ET reported a 54.2% improvement in the upper limb tremor score (43). GK pallidotomy has also been performed to treat PD, although only a few reports have described the procedure (44). The GPi is considered a high-risk target for GK radiosurgery due to its proximity to the optic tract and the risk of optic neuropathy (45). Likewise, the STN is not an advised target due to the risk of neurologic complications including hemiparesis, dysarthria, and gait disturbances (46).

### Patient considerations for SRS

A major benefit of GK radiosurgery is that it allows for precise intracranial treatment without cranial opening, eliminating the surgical risk of hemorrhage as it does not require the creation of burr holes or puncturing of brain tissue. A major drawback to GK radiosurgery for treating movement disorders is that there are often significant delays before any symptom improvement is appreciated. In previous reports, a delay of 3–6 months was noted prior to the onset of tremor suppression (42, 43). Additionally, the radiation response after GK radiosurgery can be variable. Approximately 10% of patients exhibited excess reactions, demonstrated by a high signal zone in the thalamus and surrounding area on MR images, even when the administered radiation dose was uniform (42). The delayed effect, exposure to ionizing radiation, and lack of intraoperative feedback are all disadvantages that must be considered when utilizing GK radiosurgery as a treatment for advanced PD (33). GK radiosurgery remains a good option for patients with significant medical comorbidities who cannot undergo more invasive procedures like DBS or RF (36).

### MRI-guided focused ultrasound

Early experiments investigating the neurosurgical applications of focused ultrasound demonstrated ultrasound as an effective tool for intracranial lesioning. However, craniotomies were required to avoid trajectory damage and wave distortion by the skull, and effective monitoring techniques were lacking. The subsequent development and hemispheric distribution of phased arrays have allowed for a completely transcranial procedure (47). In this procedure, an intracranial region is targeted with high-intensity focused ultrasound beams. Ablation of the target structure is achieved *via* ultrasonic mechanical energy that is absorbed within the focal target volume and converted into heat. Real-time monitoring of energy deposition and accurate targeting is made possible by MRI guidance and MR thermography (33). MRgFUS allows for accurate ablation of deep brain structures while eliminating the need for a craniotomy. An additional advancement of MRgFUS over that of other ablative procedures is that the patient can be examined after each sonication. During MRgFUS, a series of verification and low treatment sonications at low temperatures are performed, resulting in transient benefits and transient or minimal adverse effects. After clinically verifying the target with lower-temperature sonications, the temperature is then increased, resulting in a permanent lesion. MRgFUS of the VIM is currently approved for the treatment of tremors. Other targets are currently under investigation (48).

### Clinical outcomes and adverse events of MRgFUS

#### Ventral intermediate

In a study by Bond et al., 20 patients were randomized to focus ultrasound unilateral VIM thalamotomy versus 7 patients to the sham procedure for patients with medically refractory tremor-dominant PD. Hand tremor, as measured with the Clinical Rating Scale for Tremor (CRST) A + B subscores in the on-medication state, improved by 62% at 3 months. Total CRST scores improved by 44% at 3 months. The most common procedure-related AEs for all patients were finger and orofacial paresthesia. Two patients had persistent hemiparesis, and one patient had persistent mild ataxia (49).

#### Globus pallidus internus

A multicenter open-label trial investigated the efficacy and safety of MRgFUS ablation of the GPi for PD patients exhibiting levodopa responsiveness, motor fluctuations, and asymmetrical motor signs. There was a 44.5% improvement in the off-medication UPDRS-III score from baseline value at 3 months and a 45.2% improvement at 12 months. The UPDRS-IV score was used to assess the motor complications of PD. The baseline pretreatment value was reduced by 42% at 3 months, and this improvement was maintained at 12 months. Twenty persistent AEs were reported, including fine motor difficulties, dysarthria, and balance difficulties (50).

#### Subthalamic nucleus

In a pilot study conducted by Martinez-Fernandez et al., 10 patients received MRgFUS subthalamotomy for the management of asymmetric PD. In the treated hemibody, the mean UPDRS-III score improved by 53% and 47% in the off-medication and on-medication states, respectively. Persistent AE included upper limb dyskinesia in two patients (51). A randomized controlled trial was then conducted by the same group in 2020. Twenty-seven patients underwent MRgFUS subthalamotomy for asymmetric PD. The mean UPDRS-III score decreased by 52.6% in the off-medication state and by 46.5% in the on-medication state at 4 months in the active-treatment group. Several AEs in the active-treatment group were reported, including dyskinesia in the on-medication state in 6 patients, dyskinesia in the off-medication in 6 patients, weakness in 5 patients, facial weakness in 3 patients, speech disturbance in 15 patients, and gait disturbance in 13 patients. Some of these deficits persisted at 12 months in six patients (52). Consequently, the high rate of AE raises concerns for MRgFUS subthalamotomy despite the clinical benefits.

### Patient considerations for MRgFUS

MRgFUS thermal ablation may be a reasonable treatment option for patients with advanced PD who are not good candidates for more invasive techniques like DBS. As with all ablative therapies, the permanent and nonadjustable nature of MRgFUS ablation must be considered. However, during treatment, patients are clinically assessed for symptomatic improvement and adverse effects, and treatment can be adjusted as necessary. The acoustic properties of the patient's skull impact the efficiency of focusing acoustic energy on a target. These properties vary from patient to patient, and it is critical to understand the relevant ultrasound physics and limits of the technique to avoid any potential adverse effects. In general, it is recommended that patients have a skull density ratio of >0.4 to be considered candidates for this treatment. Patients with artifacts from previous cranial surgical interventions or skull shape deformations may not be suitable candidates for MRgFUS ablation (53). Other considerations are that the patient's head must be shaved, and the MRI environment and increased operative time may be uncomfortable or intolerable for claustrophobic patients. Similarly, the presence of MRI-incompatible implants must be considered (33).

### Laser-induced thermal therapy

Laser-induced thermal therapy (LITT) is an FDA-approved therapy for ablating intracranial soft tissue and brain structures including brain tumors, radiation necrosis, and epileptic foci. A laser diffusing fiber within a cooling catheter is stereotactically placed into the tissue of interest, and the laser fiber provides high-energy laser light circumferentially. The use of LITT with MRI provides real-time thermal imaging, allowing control of the ablation. MRI thermal imaging and LITT software provide real-time temperature feedback and controls, resulting in precise tissue ablation. A real-time necrotic zone and transitional zone are provided in real time. The surgeon can contour the ablation *via* either advancing or pulling back the fiber or by using multiple catheters/fibers. This therapy has been used extensively in epilepsy and brain tumors. So far, only a few case reports have been reported describing its unilateral use for pallidotomy or thalamotomy for PD (54).

## Deep brain stimulation

DBS is an FDA-approved neurosurgical procedure that delivers electrical stimulation through an electrode placed into deeper brain structures that is connected to an implanted battery source located subclavicular. Over the past two decades, DBS has become a standard of care for patients with pharmacological resistant tremors, motor fluctuations, and bothersome dyskinesias (55). The primary surgical targets are the STN and GPi. The VIM is less commonly targeted as VIM DBS is only effective for tremors and does not improve other symptoms of PD. The STN and GPi are more easily targeted with DBS than ablative procedures, which provides an added advantage to DBS.

DBS can be performed under imaging guidance alone, with microelectrode recordings, or in a combination. In addition, the patient may be alert in the setting of performing microelectrode recordings or sedated, as typically performed with imaging-alone-based surgeries or a combination. Lower hemorrhage and infection rates may be evident with general anesthesia, but higher rates of treatment-induced side effects may occur than microelectrode recording procedures. However, there may be no difference in clinical motor outcomes (56). Overall, the surgical methodology performed depends upon the experience of the center with their preferred technique.

### Clinical outcomes and adverse events of DBS

Randomized control studies of DBS to best medical treatment have demonstrated superior improvements with DBS. At 6-month postoperatively, motor symptoms in the on-medication/on-stimulation state and the quality of life improved by 20% compared with baseline, while the best medication group showed no clinical improvement (57). Furthermore, long-term reports of bilateral STN-DBS indicated that patients remain considerably improved compared with baseline (58). In a 13-year follow-up study, bilateral STN-DBS significantly improved motor symptoms in patients with early-onset PD. In the off-medication/on-stimulation state, patients had a 54% reduction in the total UPDRS-III score compared to that in the off-medication/off-stimulation state. Similarly, patients had a 48% reduction in the total UPDRS-III score in the on-medication/on-stimulation state compared to that in the on-medication/off-stimulation state. No hardware or surgical-related AEs were reported in the group of patients. Stimulation-induced dyskinesia occurred in four patients during the first 3 months of stimulation (59).

Another study retrospectively evaluated the clinical outcomes of bilateral GPi-DBS in 65 patients with PD. A 29% reduction of the total UPDRS-III score was appreciated in the off-medication state at 1-year follow-up, while no significant change was appreciated in the on-medication state. The most common AEs included gait and postural disorders, lead fractures, and dysarthria (60).

In a randomized controlled trial comparing DBS of the GPi and STN in patients with advanced PD, it was found that STN-DBS provides greater improvement in off-drug phase motor symptoms compared to GPi-DBS. Intention-to-treat analysis showed a greater reduction in the off-drug phase UPDRS-III score after 3 years in the STN group (32% reduction) compared to that in the GPi group (23% reduction) (61).

Adverse events in DBS can be separated into three categories: related to surgery, hardware, and stimulation. Adverse events related to surgery include but are not limited to infection and hemorrhage. The rate of DBS infections is generally less <7% and may necessitate complete removal of the DBS system. The use of intraoperative vancomycin powder has not been shown to decrease infection risk (62–66). The overall risk of intracranial hemorrhage is estimated to be <5%, and 1%–2% of intraventricular or intraparenchymal hemorrhage is symptomatic. Risk factors for intracranial hemorrhage include trans-sulcal trajectories and hypertension. Hardware-related adverse events occur in <2.2% of cases and include lead fracture, lead migration, and electrode malfunction (67). Stimulation side effects occur due to the electrical spread to unwanted regions that may be mediated with parameter adjustments, including focusing the volume of tissue activated with directional contacts (67). Directional leads also allow for increasing the therapeutic window, the onset of clinical benefits to that of stimulation-induced adverse effects, while resulting in less battery consumption (68).

### Patient considerations for DBS

The nonlesional and adjustable nature of DBS is among the advantages offered over other methods for neuromodulation (55). While many advantages exist, several patient-specific and ethical considerations must also be examined. DBS is a minimally invasive surgery with low incidences of infection and symptomatic hemorrhage. Additionally, DBS incurs large capital costs and requires a multidisciplinary expert team to provide optimal programming and troubleshooting for the patient. DBS can also be problematic for some in that it commits patients to a lifelong implant requiring subsequent battery (IPG) changes (55). However, due to advancing technology, battery life continues to increase and patients have the option of a rechargeable battery, thus decreasing the number of battery replacement surgeries. Patients who travel far to receive DBS surgery also now have the option to have DBS programmed remotely (69, 70). Ideal PD candidates for DBS are those with motor fluctuations and/or TSD without having other “red flags” for an alternative diagnosis. Patients with neuropsychiatric issues and multiple comorbidities may be poor candidates. Many centers exclude patients older than 70 years from DBS surgeries, although an age limit for DBS has not been defined (34).

### Lesioning procedures vs. DBS

Most clinicians favor DBS over lesioning procedures wherever the former is widely available. However, lesioning procedures are still performed in less developed countries due to financial constraints, lack of infrastructure and training, and limited follow-up care for DBS (40). Lesioning procedures are relatively cheaper and reduce postoperative care and hardware-related complications compared to DBS (71). However, lesioning procedures are not reversible, and postprocedure optimization is impossible without revision surgery. The surgical reversibility of DBS has recently been contested in the literature (72). Postmortem studies evaluating patients who have undergone DBS demonstrate pathological changes including glial scarring. It is unclear as to whether the contribution to these findings is solely due to lead insertion, chronic stimulation, or a combination (73, 74). For our review, we will consider DBS as a reversible procedure, given that the aforementioned irreversible features of DBS are not comparable to the significant irreversible changes that occur with brain lesioning procedures. Moreover, there is a lack of long-term data to support whether or not these irreversible features of DBS are truly permanent (72). The increased rate of side effects with bilateral lesions is a major limitation of lesioning procedures. These side effects include aphasia, dysarthria, dysphagia, and cognitive deficits (34). The question of morbidity with bilateral lesions has been revisited in the focused ultrasound era but has historically been problematic when treating PD patients (33, 75).

## Gene therapies

Gene supplementation therapy for PD consists of using a viral vector to deliver complementary DNA (cDNA) sequences that code for genes involved in the pathogenesis of PD. Gene therapies aim to either increase the bioavailability of DA in the nigrostriatal pathway *via* direct enhancement of enzymes involved in dopamine production or promote the health of dopaminergic neurons through maintenance and restoration of neurotrophic factors (76). The putamen is the brain region most affected by dopaminergic denervation in PD. Gene therapies aim to restore DA in the putamen *via* targeted delivery. Lack of success in earlier gene therapy clinical trials was commonly due to conservative volumes, resulting in suboptimal coverage of the putamen (77–81). Since then, the use of a pressurized infusion method in the latest clinical trials has improved the distribution of the infusate within the target (82–84).

### AAV2-hAADC

Many gene therapy studies have focused on increasing DA synthesis enzyme expression, namely, tyrosine hydroxylase (TH), L-amino acid decarboxylase (AADC), and GTP cyclohydroxylase (GCH1).

One study assessed the safety and efficacy of adeno-associated serotype 2 viral vector encoding human AADC (AAV-hAADC-2) intraputaminal infusion in advanced PD patients. At 6 months, patients demonstrated a significant improvement in both UPDRS total and UPDRS-III scores (85). In another study, advanced PD patients received low- or high-dose intraputaminal infusion of adeno-associated viral vector (AAV-hAADC). At 6 months, patients exhibited mean improvements of 36% and 28% in the off- and on-state UPDRS-III scores, respectively (86).

AADC gene therapy for PD has a favorable safety and tolerability profile. Five out of the 31 patients who received the drug across all studies reported procedure-related SAEs including four intracranial hemorrhages and one deep vein thrombosis (78, 85–88). Eight participants reported transient dyskinesia increase (87, 88).

### LV-GCH1-TH-AADC

ProSavin is a novel lentiviral vector that encodes GCH1, TH, and AADC. The phase ½ open-label, dose-escalation study has been completed, and a long-term safety and efficacy study evaluating the same patient cohort is ongoing (81, 89). OXB-102 (AXO-Lenti-PD) is an optimized version of ProSavin that expresses the same genes. A phase ½ safety and dose-escalation study is currently ongoing (90).

Patients treated with ProSavin experienced a significant reduction in the off-state UPDRS-III score, which was maintained at a 2-year follow-up (81, 89). A 21-point reduction in the UPDRS-III off-state score and a 2.2-h increase in the on time from baseline were reported in the first four patients treated with high-dose OXB-102 (90).

In total, 91% of drug-related AEs in patients treated with ProSavin were mild and/or occurred within 1-year follow-up. Acute psychosis, dyskinesia, and an unspecified nervous system disorder were among the three therapy-associated SAEs (89). No therapy-related SAEs were reported after OXB-102 treatment at 6-month follow-up (90).

### AAV2-GAD gene therapy

Loss of dopaminergic neurons in the substantia nigra results in decreased GABA input to the STN, resulting in the loss of inhibition. Infusion of muscimol (GABA) agonist into the STN has been found to improve parkinsonian symptoms temporarily. Glutamic acid decarboxylase (GAD) is the rate-limiting enzyme for GABA production, and gene therapy consisting of insertion of the GAD gene into an adeno-associated viral vector (AAV2) has been used in animal and human studies with success. The hypothesis is that delivering GAD to the STN restores GABA transmission within the STN and normalizes output from the STN. A phase I trial of 12 patients receiving a unilateral infusion of AAV2-GAD into the STN demonstrated significant improvements in motor UPDRS scores at 3 and 12 months and confirmed the safety of the therapy (91). A phase II double-blinded trial, randomizing 23 patients to sham surgery and 22 patients to bilateral STN infusion of AAV2-GAD, showed a significant improvement in motor UPDRS scores at 6 and 12 months with an associated decrease in daily dyskinesias (92, 93).

### Neurotrophic signaling restoration

PD patients demonstrate loss of endogenous neuronal growth factors in addition to dopaminergic neuron loss (76). Extensive research has been done on restoring growth factors to protect and improve the health of dopaminergic neurons. Glial cell-line derived neurotrophic factor (GDNF) and neurturin (NRTN) are two growth factors that have gained significant attention (94). Currently, only gene therapy with AAV2-GDNF is under active investigation in two clinical trials (76).

Results from the phase 1 study reported 36% and 54% increases in 18F-DOPA putaminal signal at 6- and 18-month follow-up, respectively. UPDRS scores remained stable over the course of the study, although motor symptoms in the patient cohort did not significantly worsen, as would be expected (76). AAV2-GDNF was shown to be safe and well tolerated. Scalp wound dehiscence in one patient was the only procedure-related SAE (79).

### Patient considerations for gene therapy

With genetic testing becoming more widely available, it is feasible to consider selecting patients for certain therapies based on their genotype. Patients with the advanced parkinsonian phenotype with motor fluctuations are expected to experience the greatest benefit from a gene therapy approach that aims to increase dopamine production. The unmodifiable nature of gene therapy must also be considered, particularly in patients with significantly advanced PD who are more susceptible to complications such as ICD and dopamine dysregulation syndrome. Studies investigating neurotrophic factors have emphasized the importance of targeting patients with earlier stages of PD when protecting and improving the health of dopaminergic neurons would be most beneficial (79, 80, 95).

## Cell-based therapies

Researchers are also investigating stem cell replacement as a novel way to treat diseased neuronal tissue (96). It is believed that DA cell transplantation is among the most promising cell-based therapy for PD, as replacing DA neurons into DA-depleted striatum can restore function in patients with PD (97).

Fetal ventral mesencephalon (VM) cell transplantation for the treatment of PD has been studied (97). One study reported the long-term clinical outcomes of two PD patients who received intrastriatal grafts of human fetal VM tissue. Following transplantation, one patient experienced significant motor benefits (38% improvement in the UPDRS-III score) and was able to stop levodopa treatment. This clinical benefit was sustained at 18 years post-transplantation. The other patient showed no improvement in the UPDRS-III score during the first 2 years after transplant but experienced motor function improvement 4 years postprocedure and was able to discontinue all dopaminergic medication. These motor benefits were preserved 15 years following transplantation. Graft-induced dyskinesias occurred in both patients. However, this was outweighed by the achieved clinical benefit on motor function and did not have a significant impact on overall function (98).

Mesenchymal stem cells (MSCs) have the capacity to differentiate toward dopaminergic neurons and release neurotrophic factors and have been shown to provide therapeutic benefits in animal models of PD (99). Clinical trials investigating MSC transplantation for the treatment of PD are currently underway (97).

Pluripotent stem cells (PSCs), including embryonic stem cells (ESCs) and induced pluripotent stem cells (iPSCs), can be differentiated into neurons with DA properties and are a promising source of cells for regenerative medicine. Human-induced PSCs have been shown to survive and function as midbrain dopaminergic neurons in a primate model of PD (100). Clinical trials investigating ESC and iPSC-derived cell products as therapeutic agents for PD have been started in Australia, China, and Japan (97).

## Future directions

Ongoing and future studies of advanced therapies in PD continue to expand. In ablative therapies, the potential to perform these treatments bilaterally and safely target structures with the potential of improving a greater number of parkinsonian symptoms is currently being explored. There is a large amount of ongoing research on DBS. Many research teams, including those in collaboration with DBS manufacturers, are exploring the potential for a “closed-loop” system. Biofeedback from local field potentials (the summation of synchronized cellular electrical activity) recorded from the contacts of the DBS electrode and wearable sensors may provide input to an adaptive DBS program that modifies parameters and the location of stimulation. Such systems may be better able to treat symptoms currently challenging to manage, including freezing of gait. The current location of the IPG is subclavicular connected to the DBS lead by a cable tunneled under the scalp and skin of the neck. Potential complications associated with this current hardware include skin erosion, infection, discomfort with neck and head movements, and bowstringing (migration of the IPG distal creating stretch on the connection cable). An IPG that is skull-mounted or that is the cap of the burr hole anchoring the DBS lead would reduce these potential complications and eliminate the need for a second surgery to place the IPG. Photobiomodulation, the use of infrared light delivered from a laser, may have disease-modifying effects and is also being investigated. If there are clear data demonstrating efficacy in PD human trials, it may be possible to develop technology combining photobiomodulation with DBS. Finally, future studies will hopefully progress gene therapies that either deliver neuroprotective agents or improve the function of cells or halt the neurodegenerative process. This technology will likely be based on an improved understanding of the pathophysiology of PD and potentially could become patient-specific based on the fundamental cause, including genetic for dopaminergic cell loss.

## Conclusion

The initiation of advanced therapies for PD is often considered when oral medications fail to control a patient's motor symptoms adequately. The landscape for advanced therapies for PD is rapidly evolving, and head-to-head studies comparing the efficacy and safety of treatment options are lacking. In advanced therapy selection, patient-specific considerations should be weighed along with the safety and efficacy of each treatment modality. LCIG significantly improves daily off time and on time without TSD, ranging from 1.91 to 4.4 h and 1.86 to 4.8 h, respectively (8–11). Results from key studies investigating the efficacy of LCIG are summarized in Table 1. CSAI provides daily off time reduction ranging from 1.89 to 4.2 h and an increase in daily on time without TSD of 1.97 h (24, 25). Infusion therapies are an appropriate choice for patients with levodopa-responsive PD with motor fluctuations. Patients must also be able to independently manage the pump or have a responsible caregiver who can do so (17). Initiation of LCIG or CSAI avoids an intracranial procedure. Abdominal and gastrointestinal ailments need to be considered in evaluating the placement of a PEG tube for LCIG (17). CSAI and potential future subcutaneous treatments are the least invasive, but risks of skin irritation, nodules, and infections are also a consideration (2, 26). Ablative therapies including SRS and MRgFUS allow for precise intracranial treatment without cranial opening and may be reasonable options for patients with significant comorbidities who are not good candidates for other invasive techniques. Studies evaluating the efficacy of GK thalamotomy report a 54.2% improvement in the upper limb tremor score, with up to 81.1% of patients evaluated as having good or excellent results at 24-month follow-up (42, 43). MRgFUS has been shown to provide significant improvements in motor outcomes. The results from key studies evaluating the efficacy of MRgFUS are reported in Table 2. However, drawbacks of SRS and MRgFUS must also be considered. For SRS, patients are exposed to high doses of ionizing radiation, and there are often significant delays before symptomatic improvement (42, 43). For MRgFUS, the patient's tolerability of the MRI environment and the presence of MRI-incompatible implants must be considered (33). Additionally, the acoustic properties of the patient's skull must be understood (53). For all ablative procedures, the nonreversible and nonadjustable nature of the technique must also be considered. RF ablation has largely been supplanted by DBS but remains a reasonable option in lower-resource areas (40). DBS offers the ability to interface with circuit pathology directly and is unique in that it is a nonlesional and adjustable treatment option (55). In the off-medication/on-stimulation state compared to the off-medication/off-stimulation state, bilateral STN-DBS provides reductions in the total UPDRS-III score ranging from 32% to 54% (59, 61). Similarly, bilateral GPi-DBS provides a 23%–29% reduction in the total UPDRS-III score in the off-medication state (60, 61). However, DBS poses the risk of hemorrhage and infection as it is an intracranial procedure. Additionally, DBS incurs large capital costs and commits the patient to a lifelong implant requiring subsequent battery changes (55). The advantages and drawbacks of each advanced therapy are summarized in Table 3. This review intends to help physicians understand retrospective data and patient considerations for each advanced therapy option.

**Table 1:** Key studies evaluating the efficacy of LCIG.

Study	*N*	Mean follow-up duration	Outcomes	Comments/Limitations
Olanow et al., 2014	37	12 weeks	Off time reduction: 1.91 h/day On time w/o TSD improvement: 1.86 h/day	Relatively short study duration
Buongiorno et al., 2015	72	22 months	Off time reduction: 3.8 h/day	Dropout rate of 38%
Fernandez et al., 2015	324	54 weeks	Off time reduction: 4.4 h/day On time w/o TSD improvement: 4.8 h/day	Open-label study
Antonini et al., 2017	258	640.7 days	Off time reduction (item 39): 3.9 h/day On time w/dyskinesia reduction (item 32): 1.1 h/day	Open-label study, partial data collected retrospectively
Fernandez et al., 2018	86 (efficacy subset) 262 (safety subset)	4.1 years	Off time reduction: 4 h/day On time w/o TSD improvement: 4 h/day	Open-label extension
Lopiano et al., 2019	145	2.8 years	Off time reduction: 50%	Open-label study, partial retrospective analysis

LCIG, levodopa–carbidopa intestinal gel; TSD, troublesome dyskinesia.

**Table 2:** Key studies evaluating efficacy and safety of MRgFUS.

Study	*N*	Disorder	Target	Motor outcomes (% improvement)	Adverse events	Comments/limitations
Bond et al., 2017	20	TDPD	VIM	Tremor subscore (CRST A + B) 3 months: 62% Total CRST 3 months: 44% UPDRS 3 months: 14%	1 persistent finger paresthesia 4 persistent orofacial paresthesia 2 persistent hemiparesis 1 persistent mild ataxia	20 subjects in the treatment group and 7 subjects in the sham group. The study goal was to recruit 30 subjects
Martinez-Fernandez et al., 2018	10	Asymmetric PD	STN	UPDRS-III, affected side, off 6 months: 53% UPDRS-III, affected side, on 6 months: 47% Total UPDRS-III off 6 months: 36% Total MDS-UPDRS-III on 6 months: 26% MDS-UPDRS-IV 6 months: 45%	2 upper limb dyskinesia	Open-label study
Eisenberg et al., 2020	20	PD	GPi	UDyRS 3 months: 59% 12 months: 43% MDS-UPDRS-III off 3 months: 44.5% 12 months: 45.2% MDS-UPDRS-IV 3 months: 42% 12 months: 42%	1 fine motor difficulties 3 dysarthria 1 balance difficulty	Open-label study
Martinez-Fernandez et al., 2020	27	Asymmetric PD	STN	UPDRS-III, affected side, off 4 months: 52.6% UPDRS-III, affected side, on 4 months: 46.5%	6 dyskinesia (off-medication) 6 new onset dyskinesia (on-medication) 5 weakness 3 isolated facial asymmetry 15 speech disturbance 13 gait disturbance	Two trial locations. Concern of fully blinded

MRgFUS, MRI-guided focused ultrasound; TDPD, tremor-dominant Parkinson’s disease; VIM, ventral intermediate; CRST, Clinical Rating Scale for Tremor; UPDRS, Unified Parkinson’s Disease Rating Scale; PD, Parkinson’s disease; STN, subthalamic nucleus; MDS, movement disorders society; GPi, Globus pallidus internus; UDyRS, unified dyskinesia rating scale.

**Table 3:** Advantages and drawbacks of advanced therapies.

Advanced therapy	Advantages	Drawbacks
LCIG	Adjustable and reversible No age limitation Avoids intracranial procedure	Requires surgical implantation procedure May require caregiver support Not suitable for patients with cognitive or psychiatric disturbances Risk of infection/implant issues
CSAI	Minimally invasive Adjustable and reversible Avoids intracranial procedure	May require caregiver support Not suitable for patients with cognitive or psychiatric disturbances Not suitable for patients with medical comorbidities
RF	Immediate treatment effects Less resource-intensive	Not reversible or adjustable Less predictability in lesion size and shape Surgical risk of hemorrhage
SRS	No cranial opening No surgical risk of hemorrhage Safe for patients with medical comorbidities	Not reversible or adjustable Delayed treatment response Exposure to ionizing radiation Some patients exhibit excess reactions
MRgFUS	No cranial opening Suitable alternative for patients who cannot undergo more invasive procedures	Not reversible or adjustable MRI environment may be uncomfortable/intolerable May not be suitable for patients with previous cranial surgical interventions Implants may be MRI-incompatible
DBS	Adjustable and reversible Direct interface with circuit pathology	Surgical risk of hemorrhage and infection Lifelong implant Large capital costs Requires multidisciplinary team Not suitable for patients with Cognitive/psychiatric disturbances or multiple medical comorbidities

PD, Parkinson’s disease; LCIG, levodopa–carbidopa intestinal gel; RF, radiofrequency; SRS, stereotactic radiosurgery; MRgFUS, MRI-guided focused ultrasound; DBS, deep brain stimulation.
